# Physicochemical Properties and Fiber-Drawing Ability of Tellurite Glasses in the TeO_2_-ZnO-Y_2_O_3_ Ternary System

**DOI:** 10.3390/ma15031177

**Published:** 2022-02-03

**Authors:** Clément Strutynski, Marianne Evrard, Antoine Le Gendre, Anthony Maldonado, Frédéric Désévédavy, Grégory Gadret, Jean-Charles Jules, Frédéric Smektala

**Affiliations:** Laboratoire Interdisciplinaire Carnot de Bourgogne (ICB), UMR 6303 CNRS-Université de Bourgogne Franche-Comté, Avenue Alain Savary, 21000 Dijon, France; marianne_evrard@etu.u-bourgogne.fr (M.E.); antlegendre35@gmail.com (A.L.G.); anthony_maldonado@etu.u-bourgogne.fr (A.M.); frederic.desevedavy@u-bourgogne.fr (F.D.); gregory.gadret@u-bourgogne.fr (G.G.); jean-charles.jules@u-bourgogne.fr (J.-C.J.); frederic.smektala@u-bourgogne.fr (F.S.)

**Keywords:** oxide glass, tellurite, infrared, optical fibers

## Abstract

Glasses in the TeO_2_-ZnO-Y_2_O_3_ (TZY) ternary system are examined in the present work. The vitrification domain of the chosen oxide matrix is determined and differential scanning calorimetry as well as X-ray diffraction measurements are carried out. The material characterizations reveal that Y_2_O_3_ incorporation cannot exceed 5 mol.% without causing detrimental crystallization within the glass. Optical transmission and refractive index investigations are conducted on compositions yielding fully amorphous samples. Next, the fiber drawing ability of selected yttrium-containing zinc-tellurite glasses is assessed and fiber-attenuation measurements in the mid-infrared are presented. Finally, a multimode step-index fiber is fabricated by combining a TZY cladding glass with a La_2_O_3_-based tellurite core glass. It is believed that yttrium-containing glasses could prove useful in association with other high glass transition temperature (>300 °C) TeO_2_-based materials for the design of robust optical fibers with precisely engineered refractive index profiles.

## 1. Introduction

During the past twenty years, tellurite glasses have been the focus of highly motivated research activities related to material engineering for photonic applications. Thanks to their large vitrification domain, tellurites offer a wide variety of compositions with practical advantages in different fields, ranging from the elaboration of transparent glass-ceramics [1,2], to the development of rare-earth doped amplifiers [3,4], or even for infrared (IR) transmitting optics [5]. One of the main topics where TeO_2_-based glasses have long remained interesting oxide-based materials is the development of highly nonlinear optical fiber systems [6,7,8,9] operating in the mid-infrared (MIR). However, with the ever-increasing requirements of today’s photonic devices operation conditions (for instance, higher power and harsher environment), traditional tellurite glass compositions sometime seem to be limited in terms of performances, especially because of their low transformation temperatures. This tendency is illustrated by the emergence of novel fiberizable heavy oxide glasses, such as germanate or gallate glasses, which exhibit higher glass transition temperatures and reinforced mechanical assets [10,11], and are therefore suitable for more demanding fiber usage. In this context, efforts have been put to formulate robust TeO_2_-based glass compositions with improved mechanical properties and better thermal sustainability, but which maintain optimal transmission in the MIR and significant nonlinear responses. The zinc–tellurite matrix has proven to be an excellent framework for the development of materials with high application potential in the fiber form. However, TeO_2_-ZnO binary glasses are difficult to vitrify and a third modifier or intermediate oxide generally needs to be incorporated within the matrix to produce materials with good fiber drawing ability [12]. Recently, the authors have investigated the TeO_2_-ZnO-La_2_O_3_ (TZL) vitreous system [13], which appears as an interesting system for optical fiber development. Lanthanum oxide helps to increase the glass transition temperature as well as to improve the overall mechanical properties of the glass and thermal stability. The addition of a non-alkaline or non-alkaline earth oxide also has the advantage of limiting the number of non-bridging oxygens within the glass network, which usually reduces reactivity with water. Furthermore, TZL glasses were successfully purified to produce optical fibers with an outstanding transmission of up to 4.0 µm and with minimal OH-related absorptions [14], proving the potential of lanthanide-containing zinc–tellurite glasses. Among the comparable trivalent oxides that could potentially be integrated within TeO_2_-based glass matrices, Y_2_O_3_ appears as an interesting alternative to La_2_O_3_. Yttrium is often considered as a lanthanide because of its 4d^1^5s^2^ electronic configuration, and for this reason it is deemed to have similar chemistry to that of rare-earth ions. To date, the introduction of yttrium oxide in tellurite vitreous matrices has been little explored. Yet, its addition is expected to yield interesting glass properties. Works in literature for instance indicate that Y_2_O_3_ helps to increase stability in tellurites [15] or other heavy oxide glasses [16]. Additionally, it possesses a relatively low phonon energy [17] and should therefore not hinder transmission of tellurites at longer wavelengths. Yttrium also has a smaller ionic radius as compared to lanthanum. As a result of this, the substitution of La atoms for Y atoms is supposed to significantly modify the polarizability of the considered vitreous materials. This could allow for the tailoring of the refractive index of the glass, which is useful for the development of fibers with special geometries and unique waveguiding parameters [18,19].

In this work, the physicochemical properties of glasses in the TeO_2_-ZnO-Y_2_O_3_ (TZY) ternary system are studied. At first, the vitrification domain in the TZY system is established. Differential scanning calorimetry (DSC) as well as X-ray diffraction (XRD) measurements are carried out to determine the basic properties of the fabricated materials. Following, optical transmission and compositional dependence of the refractive index are studied. Finally, the fiber drawing ability of TZY glasses is assessed through the thermal drawing of single-material as well as core/cladding preforms. It is believed those results could provide interesting insights for the development of fiber-based photonic systems in the MIR.

## 2. Materials and Methods

### 2.1. Glass Synthesis, Preform Fabrication and Fiber Drawing

Zinc–tellurite glasses in the system TeO_2_-ZnO-Y_2_O_3_ are prepared using the conventional melt-quenching technique. First, TeO_2_ (Fox Chemicals, Pfinztal, Germany 99.9%), ZnO (Alfa Aesar, Ward Hill, MS, USA 99.99%) and Y_2_O_3_ (Alfa Aesar 99.99%) in the powder form are weighed in adequate ratios and put together inside a platinum crucible. The precursor mix is then heated in an electric furnace up to 900 °C–1000 °C depending on the glass composition, and kept at this temperature for 30 min. During this stage, the hot melt is stirred manually several times to ensure good homogeneity of the final glass. Special attention is given to the homogenization of compositions with a TeO_2_ content below 70%. Subsequently, the liquid mix is poured in a parallelepipedal brass mold. Typical samples have the dimensions of 5 × 10 × 15 mm and are prepared from 5 g precursor batches. The glass is then annealed at 300 °C for 5 h and its temperature is slowly (~2 °C/min) ramped down to room temperature. A total of 60 mm long and 9 mm diameter cylindrical glass preforms used for optical fiber fabrication are prepared from ~30 g batches following the same melt-quenching procedure.

Step-index glass preforms are manufactured using the build-in casting method, which requires a special casting procedure. Detailed descriptions of the build-in casting technique are available in the literature [13,20,21]. First, 40 g batches of core and cladding glasses are prepared using the different steps described above. At the end of the synthesis time, the hot liquid cladding glass is poured inside a 16 mm diameter brass mold preheated at 350 °C. After 30 s, the mold is flipped over to remove the excess cladding glass at the center of the mold, which is still in the molten state. Finally, the core glass is subsequently poured inside the cladding glass tube formed during the previous operation. After annealing at 350 °C for 5 h, a ~ 40 mm long and 16 mm diameter core-cladding preform is obtained.

Optical fibers are manufactured from single-material or core-cladding rods using a 3 m-high drawing tower. Te glass preform is positioned inside the drawing tower furnace placed under an He gas flow (3 L/min) and ramped up (at 10 °C/min) to its softening temperature. After the glass stretching process is initiated, the drawing parameters (temperature, drum rotation speed, preform feed, fiber diameter and tension) are continuously monitored to produce fibers with adequate dimensions.

### 2.2. Physicochemical Characterizations

The glass transition temperature (*T_g_*) and crystallization temperature (*T_c_*) are determined through differential scanning calorimetry (DSC) measurements, with an accuracy of ±2 °C using a 2920 model TA Instrument apparatus. DSC thermographs of ~ 25 mg samples are registered under nitrogen gas flow between 20 and 525 °C with a 10 °C/min heating rate. The *T_g_* is taken at the inflection point of the endotherm, as obtained by taking the first derivative of the DSC curve. *T_c_* is defined as the onset of the exothermic peak.

The thermal expansion coefficient (TEC) is determined by means of a thermomechanical analyzer (TMA) 402 PC dilatometer (Netzsch, Troarn, France). Parallelepipedic glass samples with dimensions of 5 × 10 × 15 mm are heated at 10 °C/min in the 20 °C–350 °C range under air with a load of 0.2 N. The TEC is evaluated from the slope of the linear portion of the TMA curves, generally between 150 °C and 350 °C.

The density of the bulk glass samples ρ is determined through tArchimedes’ method in pure ethanol. The accuracy is better than ±0.01 g/cm^3^. The oxygen packing density (OPD) given in mol·cm^−3^ is then calculated using the following equation:

(1) $$\mathrm{OPD}=C\times \frac{\rho }{M}$$

where C is the number of oxygen atoms for each molecular unit, reduced to one total mole of oxygen, ρ is the density and M is the molecular weight.

X-ray diffraction measurements are carried out on powdered samples using an X-ray Bruker D2 Phaser XE-T (CuKα = 1.54184 Å radiation) diffractometer. Diffractograms are collected over the 20–80° 2θ range with a 0.02017° angle step. The crystal phases are then identified using X′Pert High Score Plus software together with the International Centre for Diffraction Data (ICDD) database.

### 2.3. Optical Properties

The UV-visible-MIR transmission spectra of the glasses are recorded using an UV-vis-NIR spectrometer (Lambda 900, Perkin-Elmer, Villebon-sur-Yvette, France) in the 200–3300 nm range and using a Fourier transform infrared (FTIR) spectrometer (Spectrum One, Perkin-Elmer, Villebon-sur-Yvette, France) in the 1500–7000 nm range. The measurements are carried out on few millimeters optically polished glass slabs.

The refractive index of the glass samples is measured at 4 different wavelengths (542, 632, 1064 and 1550 nm) on carefully polished slabs. The measurements are carried out using a homemade prism (TiO_2_) refractometer with an estimated error of 10^−3^. The refractive index dispersion is then fitted as a function of the wavelength by the two-pole Sellmeier equation:

(2) $$n^{2}\left(\lambda \right)\;=A+\frac{{B\lambda }^{2}}{\lambda^{2}-C^{2}}$$

with λ, the wavelength and A, B and C, the Sellmeier coefficients, obtained with a least-square fitting procedure.

Optical losses measurements of single-index and step-index fibers are carried out using the cutback method on several meters-long (2–3 m) samples. An external 100 W halogen lamp emitting from 0.4 to 5.2 µm is coupled into the fiber by means of a mirror objective, while the output signal is recorded using an InSb photodetector linked to a FTIR spectrometer (Nicolet 6700, Fisher Scientific, Illkirch, France) operating in the 1–5 µm range.

## 3. Results and Discussion

### 3.1. TZY Vitreous Domain

**Figure 1:**
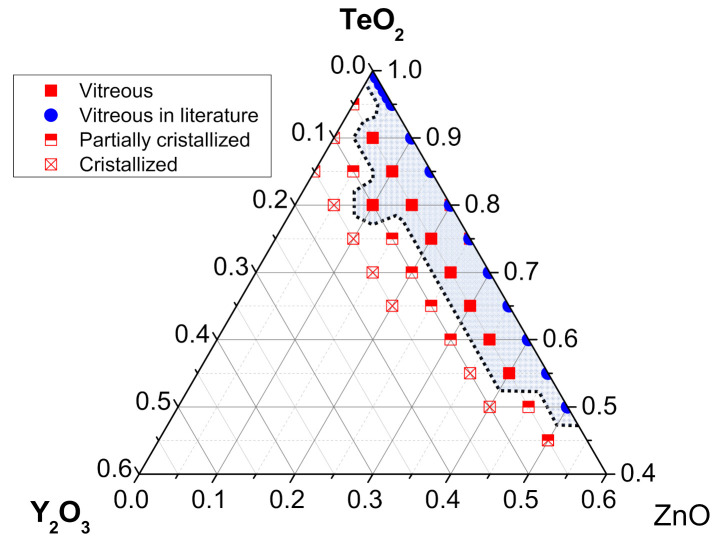
TeO_2_-ZnO-Y_2_O_3_ ternary diagram.

As a first experiment, 5 g test batches in the TeO_2_-ZnO-Y_2_O_3_ system are synthesized in order to establish the glass-forming TZY compositions. For clarity, the different glass samples are named accordingly to their TeO_2_ and Y2O_3_ concentrations while the zinc oxide content is implicit in the notation. In that case, the composition 80 TeO_2_-10 ZnO-10 Y_2_O_3_ is for instance noted (T80Y10). Overall, the synthesis of TZY glasses is more complex than that of TeO_2_-ZnO-La_2_O_3_ glasses. Additional attention is needed during melting, and stirring or mixing operations are required as well as quenching–grinding–remelting procedures to produce homogeneous samples, especially for low TeO_2_ content compositions. As a result of this, the nature (amorphous, crystallized or partially-crystallized) of each sample is determined through X-ray diffraction analysis and reported on the ternary diagram, shown in Figure 1.

**Figure 2:**
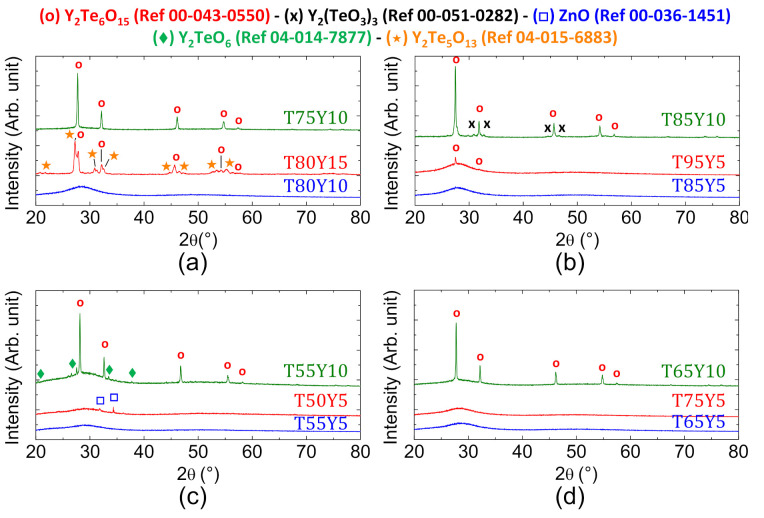
X-ray diffraction diagrams of (**a**) the T75Y10, T80Y15 and T80Y10 samples; (**b**) the T85Y10, T95Y5 and T85Y5 samples; (**c**) the T55Y10, T50Y5 and T55Y5 samples and (**d**) the T65Y10, T75Y5 and T65Y5 samples.

A sample is considered fully amorphous when no distinct peak is visible on its diffractogram. Those first characterizations reveal that Y_2_O_3_ incorporation cannot exceed 5 mol.% without causing detrimental crystallization within the glass, with the exception of one composition. Oxides ratios for this particular composition are (in mol.%) 80 TeO_2_-10 ZnO-10 Y_2_O_3_. In a similar way to TZL glasses, TeO_2_ content must be greater than 55 mol.% in order to form completely vitreous samples. The X-ray diffractograms of the selected test batches are plotted in Figure 2. The crystalline phases identified in the materials are in good agreement with the information available in the literature [22].

### 3.2. Thermal Properties

**Figure 3:**
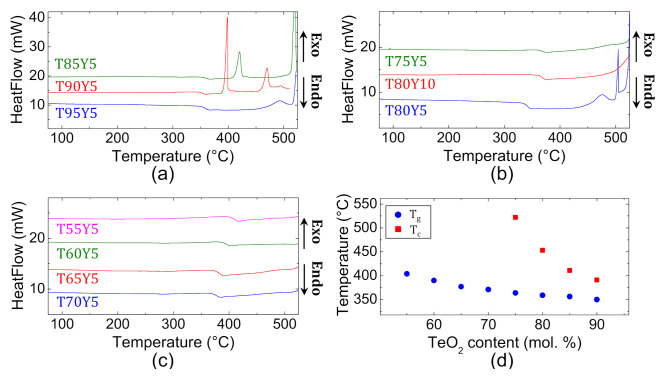
DSC thermographs of (**a**) the T85Y5, T90Y5 and T95Y5 samples; (**b**) the T75Y5, T80Y10 and T80Y5 samples and (**c**) the T55Y5, T60Y5, T65Y5 and T70Y5 samples. (**d**) Compositional dependence of glass transition temperature T_g_ and onset crystallization temperature T_c_ in the glass series (100 − x) TeO_2_-x ZnO-5 Y_2_O_3_ (with x = 0, 5, 10, 15, 20, 25, 30, 35, 40).

Thermal properties of test batches yielding fully amorphous samples are studied through differential scanning calorimetry (DSC). The DSC curves of the different glasses are plotted in Figure 3. Thermograms of samples with TeO_2_ content below 70 mol.% (Figure 3c) have *T_g_* endothermic signatures below 400 °C and do not exhibit any exothermic event on the considered temperature measuring range (20–525 °C), which attests for the good thermal stability of those compositions. The glass transition temperatures *T_g_*, the onset crystallization temperature *T_c_* as well as the parameter of thermal stability *ΔT* = *T_c_*−*T_g_* are recapped in Table 1. The glass transition temperature of the different samples varies from ~350 °C to ~400 °C, which makes TZY glasses thermally compatible with their lanthanum-containing counterparts as they possess similar *T_g_* ranges [13]. The compositional dependence of the glass transition temperature *T_g_* as well as the onset crystallization temperature *T_c_* is plotted in Figure 3d. From this graph, it is obvious that *T_g_* monotonously decreases with the increasing tellurium oxide content. Due to its shorter length, the Zn–O bond has a higher binding energy as compared to the Te–O bond. This is illustrated in particular by the fusion temperature of zinc oxide (1975 °C), which is significantly greater than that of tellurium oxide (733 °C) [23]. As a consequence, compositions with higher ZnO content possess higher glass transition temperatures. Additionally, for a fixed molar percentage of TeO_2_, the glass transition temperature increases with the amount of Y_2_O_3_ incorporated in the glass: the composition (in mol.%) 80 TeO_2_-10 ZnO-10 Y_2_O_3_ has a *T_g_* of 363 °C when the composition 80 TeO_2_-15 ZnO-5 Y_2_O_3_ has a *T_g_* of 358 °C. As previously mentioned, this can be explained by the binding energies of the oxides involved in the glass, as Y–O has a stronger binding energy (Y_2_O_3_ has a fusion temperature of 2425 °C) as compared to Zn–O. At the same time, the thermal stability also decreases with the TeO_2_ content. Empirically, glasses are considered suitable for fiber drawing when the difference Δ*T* = *T_c_*−*T_g_* is greater than 100 °C, which is not the case here for the compositions with TeO_2_ content exceeding 80 mol.%. Finally, density of the different glasses is measured by Archimedes’ method and the results are gathered in Table 1. The density decreases when substituting TeO_2_ for ZnO, which is usually the case when replacing a high molar mass oxide (159.6 g/mol for tellurium oxide) by another with a lower molar mass (81.4 g/mol for zinc oxide).

### 3.3. Optical Properties

Additionally, the optical characterizations of the different glasses are performed. The refractive index of the different samples is measured at 543.2 nm, 632.8 nm, 1064 nm and 1550 nm. The results are reported in Table 2. The refractive index range of the present TZY glasses is comparable to that of the zinc–tellurite glasses already reported in the literature [24,25,26,27]. At 1550 nm, it ranges from 1.939 to 2.062 which is slightly lower than that of lanthanum-containing glasses [13]. For the identical incorporation of either La_2_O_3_ or Y_2_O_3_ in the zinc–tellurite matrix, yttrium-based materials have a lower refractive index. For instance, the glass of composition (in mol.%) 80 TeO_2_-15 ZnO-5 Y_2_O_3_ has a refractive index of 2.024 when the composition 80 TeO_2_-15 ZnO-5 La_2_O_3_ has a refractive index of 2.037. This difference can be partly attributed to the greater polarizing effect of yttrium ions on oxygens because of their smaller ionic radius (90.0 pm) as compared to La ions (103.2 pm) [28]. As a consequence, oxygen atoms, which are more polarized by yttrium ions, are less polarizable by an external electromagnetic field. The polarizability of the glass is decreased for Y_2_O_3_-based materials, which explains the refractive index values measured for the TZY samples.

**Figure 4:**
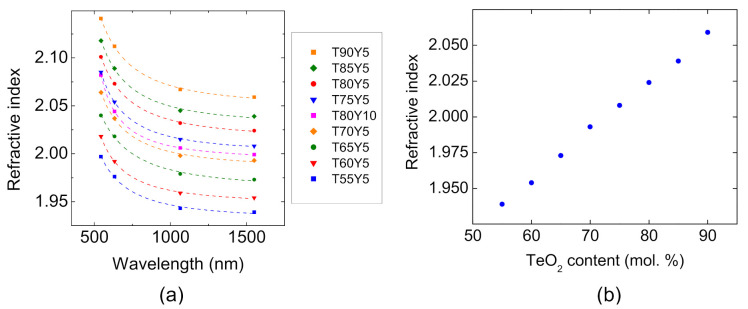
(**a**) Refractive indices of the glasses considered in this work measured at 543.3, 632.8, 1064 and 1550 nm. The curves in dashed lines correspond to the Sellemeier fit of the experimental values. (**b**) Compositional dependence of the refractive index measured at 1550 nm.

Refractive index control is of high interest for the design of optical fibers with particular waveguiding parameters. The evolution of the refractive index as a function of wavelength is plotted in Figure 4a for the different Yttrium containing zinc–tellurite glasses. Additionally, the experimental data are fitted to a two-pole Sellmeier equation (see Equation (2)).

The Sellemeier parameters, A, B and C, are obtained with a least-square fitting procedure and are gathered in Table 3. It is worth mentioning that the accuracy of the fitting procedure is limited to wavelengths within the experimental measurement range, namely between 543.5 nm and 1550 nm, and the refractive index values cannot be extrapolated precisely beyond this domain. The evolution of the refractive index measured at 1.55 µm as a function of the tellurium oxide content is plotted in Figure 4b.

**Figure 5:**
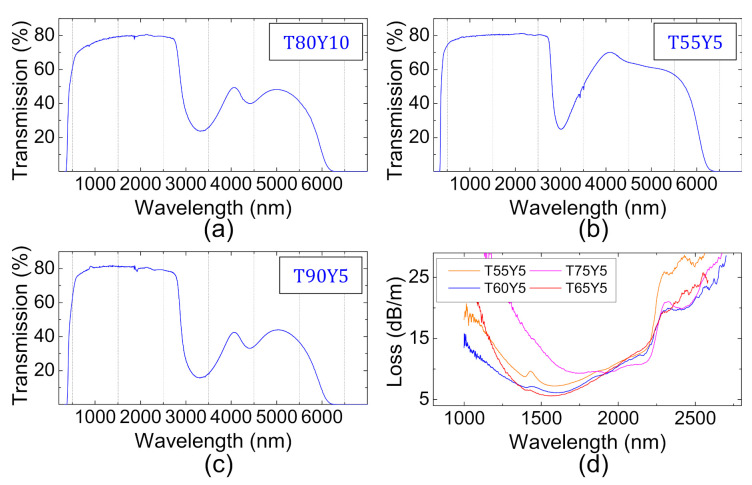
Bulk glass transmission from UV up to the mid-IR for a (**a**) 4.38 mm thick T80Y10 sample, (**b**) 4.47 mm thick T55Y5 sample and (**c**) 4.42 mm thick T90Y5 sample. (**d**) The optical attenuation curve of various single-material TZY glass fibers.

The refractive index variation follows a linear increasing trend with the concentration of TeO_2_ in the glass. This can be well explained by the related increasing of the OPD with the tellurium content, at a fixed yttrium content (see Table 1). Next, the optical transmission of selected TZY compositions are measured from UV up to the MIR. The recorded curves are shown in Figure 5a–c for the T80Y10, T55Y5 and T90Y5 glasses, respectively. Those compositions are investigated due to their extreme positions on the TeO_2_-ZnO-Y_2_O_3_ ternary diagram. They exhibit a typical tellurite glass transmission ranging from ~350 nm up to ~6000 nm in the infrared. TeO_2_-rich compositions have a slightly reduced transmission at shorter wavelengths. The UV edge is indeed 349 nm, 389 nm and 402 nm for the T55Y5, T80Y10 and T90Y5 samples, respectively. For comparison purposes, the UV transmission edge is arbitrarily defined here for the ~4 mm thick samples as the wavelength at which the absorption of the glass is equal to 4 cm^−1^.

The shift of the UV edge, which causes a yellow coloration in tellurite glasses while they are initially colorless [29,30], is usually attributed to the presence of platinum within the glass matrix. This pollution originates from the crucible used for synthesis. Pt dissolution into the glass occurs during the melting procedure due to corrosive reactions between the hot liquid glass and the crucible. It is promoted by the oxidizing conditions during glass synthesis, and can be reduced or controlled depending on the redox conditions of the synthesis [30]. This phenomenon is for instance observed in Pt crucible-grown TeO_2_ single crystals [31] or in phosphate glasses melted in Pt crucibles [32]. Platinum solubility also strongly depends on the glass composition, as demonstrated in silver-containing zinc–tellurite vitreous materials [25]. Here, it is highly likely that Pt solubility is greater for compositions with a higher tellurium oxide concentration, which explains their reduced transmission in the UV. Further characterizations are however necessary to fully apprehend platinum pollution in TZY glasses. Concerning the transmission in the MIR, all 3 samples show strong OH-related absorptions at ~3400 nm and ~4300 nm, due to the synthesis, which is performed under room atmosphere. The low TeO_2_ content T55Y5 sample however exhibits different absorption band structures as compared to the two other samples. The absorption band usually located around 3400 nm and generally attributed to a combination of free OH absorption (centered at 3030 nm) and weakly hydrogen-bonded Te–OH absorption (centered at 3260 nm) [33,34,35] is strongly dissymmetric with a maximum centered at 3000 nm. The contribution of H-bonded Te–OH groups is in fact highly reduced for this band. Regarding the absorption usually located around 4400 nm in tellurite glasses and attributed to strongly hydrogen-bonded Te–OH groups, it is almost non-existent for the T55Y5 sample. These observations indicate that in low TeO_2_ content TZY glasses, the number of strongly or weakly hydrogen-bonded T–-OH groups is significantly reduced, and that OH ions mainly exist as free hydroxyl groups. In oxide glasses that contain a modifier, H-bonds (weak or strong) form preferably with non-bridging oxygens (NBOs) [36]. From a structural point of view, it is safe to consider that the amount of NBOs is significantly lower within the T55Y5 glass as compared to the T90Y5 glass. That observation can be related to the low content of TeO_2_ glass former in T55Y5 glass, leading the intermediate ZnO to act as a glass former in this composition, when it essentially acts as a glass modifier in the T90Y5 composition. Additionally, the oxygen packing density (OPD) calculation is helpful to understand the compositional dependence of the H-bonded Te–OH group’s concentration within the glass. The OPD, given in Table 1, provides an insight concerning the oxygen concentration inside a given vitreous matrix. According to Scholze et al. [36], the concentration of H-bonded hydroxyl groups in silicates depends on the average distance between oxygen atoms, in other words, it depends on the amount of oxygens per unit volume. Here, in the TeO_2_-ZnO-Y_2_O_3_ system, the OPD decreases when the tellurium oxide content decreases (see Table 1). OPD is 6.92 × 10^−2^, 6.84 × 10^−2^ and 6.72 × 10^−2^ mol.cm^−3^ for T90Y5, T80Y10 and T55Y5, respectively. The lower the TeO_2_ concentration, the lower the oxygen packing density, and the higher the average distance between oxygens atoms. As a result, H-bonds are less likely to form in low tellurium oxide content TZY glasses. Concerning the MIR transmission limit, TZY samples possess a similar multi-phonon edge as compared to TZL glasses [13]. In tellurite glasses, the transmission towards longer wavelengths is limited, in first approximation, by the oxide contained in the vitreous matrix with the highest phonon energy. In order to take advantage of the full transmission window of tellurites, it is therefore preferable to add intermediate or modifier oxides with phonon energies lower than that of TeO_2_ (which is 650 cm^−1^). The addition of Nb_2_O_5_ or WO_3_, which have phonon energies of, respectively 860 cm^−1^ and 925 cm^−1^, would, for instance, shift the IR transmission edge towards shorter wavelengths [37]. Yttrium oxide has a phonon of energy of 590 cm^−1^ [17] and is therefore not expected to hinder zinc-tellurite glass transmission in the MIR, which is verified here by the bulk transmission measurements shown in Figure 5a–c.

Finally, the fiber drawing ability of TZY materials was assessed. Based on the DSC measurements presented in the previous section, T55Y5, T60Y5, T65Y5 and T75Y5 single-material glass rods are fabricated and thermally elongated into meters of optical fibers. Optical attenuation loss measurements are then performed on those fibers and the results are shown in Figure 5d. All the different compositions possess a similar fiber-transmission, which extends up to approximately 2700 nm in the MIR. A minimum loss of ~6.1 dB/m located around 1500–1600 nm is attained for the best attenuation curves. The infrared transmission edge is typical of fibers made from glasses synthesized under room atmosphere and without particular care. Still, unpurified TZY glass fibers propagate light further in the infrared than their alkaline oxide and alkaline-earth oxide containing counterparts, which are limited to ~1800 nm [7,38]. The fiber transmission can be significantly enhanced by using higher quality precursors (purity of 99.999%) to decrease the background losses and by implementing a purification protocol in order to extent transmission towards the infrared [14].

### 3.4. Multi-Mode Step-Index Fiber Fabrication

In this final section, an yttrium-containing zinc–tellurite composition is selected and associated with a TZL glass to design a step-index multi-mode optical fiber. As described in Section 3.3, TZY compositions possess lower refractive indices as compared to their TZL counterparts, but with similar glass transitions temperatures, and are thus suitable to be used as cladding. This also means that TZY and TZL glasses can be associated with manufacture fibers with higher numerical aperture than what would be available from association of glasses within the same ternary diagram. Here, the 72 TeO_2_-20 ZnO-8 La_2_O_3_ composition (T72L8) is selected as the core material and the T60Y5 glass as the cladding material. Their main properties are reported in Table 4. Both glasses possess good thermal stability as well as similar glass transitions temperatures and thermal expansion coefficients, which means they can be simultaneously co-drawn into optical fibers without risking any major recrystallization or mechanical failure during the thermal process. The glasses have a refractive index difference of 5.2 × 10^−2^ at 1550 nm, which is significantly higher than the 1.9 × 10^−2^ reported for TZL/TZL associations [13,14].

**Figure 6:**
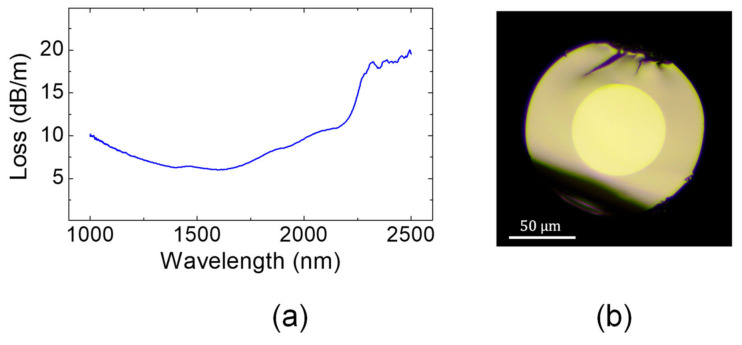
(**a**) Optical attenuation curve and (**b**) cross-sectional view of the multimode T72La8/T60Y5 fiber, which has a numerical aperture of 0.52.

A core-cladding preform is fabricated from those two glasses by the build-in casting technique and is subsequently elongated into meters of fiber. A cross-sectional view of the fabricated fiber is shown in Figure 6b. Given the refractive indices of the core and the cladding, the numerical aperture of the fabricated step-index fiber is ~0.45 at 1550 nm. The diameter of the core is ~62 µm and the external diameter is ~120 µm, which means the ratio between the core and the cladding is approximately 0.52. No major defects are visible on the micrograph, and the interface between the two glasses is of good quality. This means that no interdiffusion or recrystallization occur between the TZL and TZY materials during the thermal elongation process. The MIR transmission properties of the large-cores step-index fiber is then evaluated through cutback measurements. The plot of the measured absorption spectrum is presented in Figure 6a. The fiber exhibits a typical tellurite glass fiber transmission with a minimum loss of ~6.8 dB/m in the 1500–1600 nm range, and light propagation is possible by up to 2500 nm in the MIR. In fact, the attenuation curve of the core-cladding fiber is similar to that of single-material fibers presented in Figure 5d, which means that the fabrication method used for the production of the step-index structure (build-in casting) did not cause any additional losses. In the end, relevance of yttrium-containing zinc–tellurite glasses for the development of MIR-transmitting fibers is demonstrated in the present study. They could, for instance, be associated with other robust tellurite compositions for the design of waveguides with advanced dispersion parameters, such as w-type fibers or others. As already stated, the attenuation level attained here can significantly be improved through purification procedures [14].

## 4. Conclusions

The potential of glasses in the TeO_2_-ZnO-Y_2_O_3_ (TZY) system for fiber fabrication was investigated in this work. From a general standpoint, the synthesis of yttrium-containing zinc–tellurite glasses is trickier than that of lanthanum-based glasses as they possess a narrower vitrification domain; only compositions with less than 5 mol. Y_2_O_3_ produce fully amorphous materials. The synthesis also requires higher temperatures, which is especially true for the manufacturing of glass preforms from low TeO_2_ content glasses that are made from larger precursor batches. Still, the TZY system offers an interesting matrix for the development of robust vitreous materials suitable for the manufacturing of MIR-transmitting fibers. The refractive index of TZY compositions ranges from 1.939 to 2.062 at 1.55 µm, which is similar to that of other zinc–tellurite materials reported in the literature. Thermal investigations revealed that TZY materials exhibit relatively elevated glass transition temperatures varying from 350 °C to 404 °C. It was also evidenced that compositions with TeO_2_ content between 55 and 80 mol.% possess good thermal stability (ΔT > 100 °C) and are therefore suitable for fiber-drawing. Furthermore, optical fibers were manufactured from selected compositions and cutback measurements were carried out. The fabricated fibers possessed background attenuation level of ~6 dB/m, which is similar to that of other tellurite glass fibers when prepared with no particular purification protocol. Those glasses could be useful in association with other high *T_g_* tellurite glasses for the design of fibers with precisely engineered dispersion profiles, which are required for the triggering of specific nonlinear dynamics.

## Figures and Tables

**Table 1:** Physico-chemical properties of glass forming compositions in the TeO_2_-ZnO-Y_2_O_3_ ternary system. Glass transition temperature (T_g_), onset crystallization temperature (T_c_), thermal stability ΔT = T_c_−T_g_, density ρ and oxygen packing density (OPD).

Name	TeO_2_	ZnO	Y_2_O_3_	T_g_ (±2 °C)	T_c_ (±2 °C)	ΔT (±4 °C)	ρ (±0.02 g·cm^−3^)	OPD (10^−2^ mol·cm^−3^)
T90Y5	90	5	5	350	395	45	5.50	6.92
T85Y5	85	10	5	356	411	55	5.47	6.88
T80Y5	80	15	5	358	453	94	5.46	6.86
T75Y5	75	20	5	364	522	158	5.45	6.85
T70Y5	70	25	5	371	>525	>154	5.44	6.83
T65Y5	65	30	5	377	>525	>148	5.42	6.80
T60Y5	60	35	5	390	>525	>135	5.38	6.75
T55Y5	55	40	5	404	>525	>121	5.36	6.72
T80Y10	80	10	10	363	505	143	5.42	6.84

**Table 2:** Refractive indices of the glasses considered in this work measured at 543.3, 632.8, 1064 and 1550 nm.

Name	Refractive Index (±0.001)			
	543.5 nm	632.8 nm	1064 nm	1550 nm
T90Y5	2.141	2.112	2.067	2.059
T85Y5	2.118	2.089	2.045	2.039
T80Y5	2.101	2.073	2.032	2.024
T75Y5	2.085	2.054	2.015	2.008
T70Y5	2.064	2.037	1.998	1.993
T65Y5	2.040	2.018	1.979	1.973
T60Y5	2.018	1.992	1.959	1.954
T55Y5	1.997	1.976	1.943	1.939
T80Y10	2.082	2.044	2.006	1.999

**Table 3:** Sellemeier parameters A, B and C obtained with a least-square fitting procedure for each of the TZY glass samples.

Name	Sellmeier Parameters		
	A	B	C (nm)
T90Y5	3.20823	0.99194	287.327
T85Y5	3.36785	0.75209	311.345
T80Y5	3.25829	0.80467	299.770
T75Y5	3.51729	0.48671	349.622
T70Y5	3.37904	0.56144	327.705
T65Y5	2.68282	1.17411	247.206
T60Y5	3.41637	0.37811	353.897
T55Y5	3.17294	0.55926	304.957
T80Y10	3.6632	0.31054	398.495

**Table 4:** Properties of the different glasses involved in the fabrication of a multimode step-index tellurite fiber.

Name	T_g_ (±2 °C)	T_c_ (±2 °C)	ΔT (±4 °C)	TEC (10^−5^·C^−1^)	n_1550nm_ [±0.001]
T72L8 (core)	387	>525	>138	1.933	2.006
T60Y5 (cladding)	390	>525	>135	1.894	1.954

## Data Availability

Not applicable.

## References

[1] Laval J.-P., Duclère J.-R., Couderc V., Allix M., Genevois C., Sarou-Kanian V., Fayon F., Coulon P.-E., Chenu S., Colas M. (2019). Highly Transparent Fluorotellurite Glass-Ceramics: Structural Investigations and Luminescence Properties. Inorg. Chem..

[2] Santos Barbosa J., Batista G., Danto S., Fargin E., Cardinal T., Poirier G., Castro Cassanjes F. (2021). Transparent Glasses and Glass-Ceramics in the Ternary System TeO_2_-Nb_2_O_5_-PbF_2_. Materials.

[3] Dorofeev V.V., Koltashev V.V., Motorin S.E., Plekhovich A.D., Kim A.V. (2021). Thermal, Optical, and IR-Emission Properties of Extremely Low Hydroxyl TeO_2_-WO_3_-Bi2O_3_-La_2_O_3_-xEr_2_O_3_ Glasses for Mid-Infrared Photonics. Photonics.

[4] Mori A., Ohishi Y., Sudo S. (1997). Erbium-doped tellurite glass fibre laser and amplifier. Electron. Lett..

[5] Linganna K., In J.-H., Kim S.H., Han K., Choi J.H. (2020). Engineering of TeO_2_-ZnO-BaO-Based Glasses for Mid-Infrared Transmitting Optics. Materials.

[6] Anashkina E.A., Dorofeev V.V., Skobelev S.A., Balakin A.A., Motorin S.E., Kosolapov A.F., Andrianov A.V. (2020). Microstructured Fibers Based on Tellurite Glass for Nonlinear Conversion of Mid-IR Ultrashort Optical Pulses. Photonics.

[7] Ebendorff-Heidepriem H., Kuan K., Oermann M.R., Knight K., Monro T.M. (2012). Extruded tellurite glass and fibers with low OH content for mid-infraredapplications. Opt. Mater. Express.

[8] Liao M., Chaudhari C., Qin G., Yan X., Suzuki T., Ohishi Y. (2009). Tellurite microstructure fibers with small hexagonal core for supercontinuum generation. Opt. Express.

[9] Kedenburg S., Steinle T., Mörz F., Steinmann A., Nguyen D., Rhonehouse D., Zong J., Chavez-Pirson A., Giessen H. (2016). Solitonic supercontinuum of femtosecond mid-IR pulses in W-type index tellurite fibers with two zero dispersion wavelengths. APL Photonics.

[10] Wen X., Tang G., Yang Q., Chen X., Qian Q., Zhang Q., Yang Z. (2016). Highly Tm^3+^ doped germanate glass and its single mode fiber for 2.0 μm laser. Sci. Rep..

[11] Strutynski C., Calzavara F., Guerineau T., Loi L., Laberdesque R., Rampnoux J.-M., Morency S., Ledemi Y., Petit Y., Dussauze M. (2021). Heavy-oxide glasses with superior mechanical assets for nonlinear fiber applications in the mid-infrared. Opt. Mater. Express.

[12] El-Mallawany R.A.H. (2016). Tellurite Glasses Handbook.

[13] Maldonado A., Evrard M., Serrano E., Crochetet A., Désévédavy F., Jules J.C., Gadret G., Brachais C.H., Strutynski C., Ledemi Y. (2021). TeO_2_-ZnO-La_2_O_3_ tellurite glass system investigation for mid-infrared robust optical fibers manufacturing. J. Alloy. Compd..

[14] Evrard M., Combes T., Maldonado A., Desevedavy F., Gadret G., Strutynski C., Jean-charles J., Brachais C.-H., Smektala F. (2021). TeO_2_–ZnO-La_2_O_3_ tellurite glasses purification for mid-infrared optical fibers manufacturing. Opt. Mater. Express.

[15] Feng X., Tanabe S., Hanada T. (2001). Spectroscopic Properties and Thermal Stability of Er ^3+^ -Doped Germanotellurite Glasses for Broadband Fiber Amplifiers. J. Am. Ceram. Soc..

[16] Cui S., Li J., Zeng H., Zhang L. (2021). Regulation of Y_2_O_3_ on glass stability of Ga_2_O_3_-rich oxyfluoride glasses. J. Non. Cryst. Solids.

[17] Repelin Y., Proust C., Husson E., Beny J.M. (1995). Vibrational Spectroscopy of the C-Form of Yttrium Sesquioxide. J. Solid State Chem..

[18] Strutynski C., Froidevaux P., Désévédavy F., Jules J.-C.J.-C., Gadret G., Bendahmane A., Tarnowski K., Kibler B., Smektala F. (2017). Tailoring supercontinuum generation beyond 2 μm in step-index tellurite fibers. Opt. Lett..

[19] Nguyen H.P.T., Tong T.H., Saini T.S., Luo X., Suzuki T., Ohishi Y. (2019). Highly coherent supercontinuum generation in a tellurite all-solid hybrid microstructured fiber pumped at 2 μm. Appl. Phys. Express.

[20] Clarke K., Ito Y. (1992). Manufacture of fluoride glass preforms. J. Non. Cryst. Solids.

[21] Strutynski C., Picot-Clemente J., Lemiere A., Froidevaux P., Désévédavy F., Gadret G., Jules J.-C.J.-C., Kibler B., Smektala F., Picot-Clémente J. (2016). Fabrication and characterization of step-index tellurite fibers with varying numerical aperture for near- and mid-infrared nonlinear optics. J. Opt. Soc. Am. B.

[22] Noguera O., Jouin J., Masson O., Jancar B., Thomas P. (2012). Phase formation and crystal structure determination in the Y_2_O_3_–TeO_2_ system prepared in an oxygen atmosphere. J. Eur. Ceram. Soc..

[23] Samsonov G.V. (2013). The Oxide Handbook.

[24] Manikandan N., Ryasnyanskiy A., Toulouse J. (2012). Thermal and optical properties of TeO2–ZnO–BaO glasses. J. Non. Cryst. Solids.

[25] Boiruchon D., Desevedavy F., Chenu S., Strutynski C., Smektala F., Gadret G., Dussauze M., Jubera V., Messaddeq Y., Cardinal T. (2019). Investigation of the Na_2_O/Ag_2_O ratio on the synthesis conditions and properties of the 80TeO_2_–10ZnO–[(10−x)Na_2_O–xAg_2_O] glasses. J. Non. Cryst. Solids.

[26] De Clermont-Gallerande J., Saito S., Colas M., Thomas P., Hayakawa T. (2021). New understanding of TeO_2_–ZnO–Na_2_O ternary glass system. J. Alloy. Compd..

[27] Aida K., Komatsu T., Dimitrov V. (2001). Thermal stability, electronic polarisability and optical basicity of ternary tellurite glasses. Phys. Chem. Glas..

[28] Shannon R.D., Prewitt C.T. (1969). Effective ionic radii in oxides and fluorides. Acta Crystallogr. B Struct. Crystallogr. Cryst. Chem..

[29] Dorofeev V.V., Moiseev A.N., Churbanov M.F., Snopatin G.E., Chilyasov A.V., Kraev I.A., Lobanov A.S., Kotereva T.V., Ketkova L.A., Pushkin A.A. (2011). High-purity TeO_2_–WO_3_–(La_2_O_3_,Bi_2_O_3_) glasses for fiber-optics. Opt. Mater..

[30] Désévédavy F., Strutynski C., Lemière A., Mathey P., Gadret G., Jules J., Kibler B., Smektala F. (2020). Review of tellurite glasses purification issues for mid-IR optical fiber applications. J. Am. Ceram. Soc..

[31] Mangin J., Veber P. (2008). PtTe2: Potential new material for the growth of defect-free TeO_2_ single crystals. J. Cryst. Growth.

[32] Karabulut M., Marasinghe G.K., Click C.A., Metwalli E., Brow R.K., Booth C.H., Bucher J.J., Shuh D.K., Suratwala T.I., Campbell J.H. (2004). XAFS Investigation of Platinum Impurities in Phosphate Glasses. J. Am. Ceram. Soc..

[33] O’Donnell M.D., Miller C.A., Furniss D., Tikhomirov V.K., Seddon A.B. (2003). Fluorotellurite glasses with improved mid-infrared transmission. J. Non. Cryst. Solids.

[34] Ryskin Y.I. (1971). The structure and infrared spectra of acid silicates. Izv. Akad. Nauk. SSSR Neorg. Mater..

[35] Ryskin Y.I., Stavitskaya G.P. (1960). Spectroscopic investigation of the hydrogen bond in acid silicatesand phosphates. Opt. Spectrosc..

[36] Scholze H. (2012). Glass: Nature, Structure, and Properties.

[37] O’Donnell M.D., Richardson K., Stolen R., Seddon A., Furniss D., Tikhomirov V.K., Rivero C., Ramme M., Stegeman R., Stegeman G. (2007). Tellurite and Fluorotellurite Glasses for Fiberoptic Raman Amplifiers:Glass Characterization, Optical Properties, Raman Gain, PreliminaryFiberization, and Fiber Characterization. J. Am. Ceram. Soc..

[38] Savelii I., Desevedavy F., Jules J.-C., Gadret G., Fatome J., Kibler B., Kawashima H., Ohishi Y., Smektala F. (2013). Management of OH absorption in tellurite optical fibers and relatedsupercontinuum generation. Opt. Mater..

